# Contribution of a synchronic teleneurology program to decrease the patient number waiting for a first consultation and their waiting time in Chile

**DOI:** 10.1186/s12911-020-1034-2

**Published:** 2020-02-05

**Authors:** Freddy Constanzo, Paula Aracena-Sherck, Juan Pablo Hidalgo, Lorena Peña, Mery Marrugo, Jonathan Gonzalez, Gerardo Vergara, Cristóbal Alvarado

**Affiliations:** 1 Neurology Unit, Hospital Las Higueras, Alto Horno 777, Talcahuano, Chile; 2 Medical Program in Adult Neurology, School of Medicine, Universidad Católica de la Santísima Concepción, Concepción, Chile; 3 Department of Science, School of Medicine and Science, Universidad San Sebastián, Concepción, Chile; 4 Department of Statistics, School of Sciences, Universidad del Bío-Bío, Concepción, Chile; 5 Unit of Teleprocesses, Hospital Las Higueras, Talcahuano, Chile; 6 Department of Basic Sciences, School of Medicine, Universidad Católica de la Santísima Concepción, Concepción, Chile

**Keywords:** Telemedicine, Teleneurology, Adult neurology

## Abstract

**Abstract:**

Backround

There is a shortage of medical specialists in Chile, including neurologists; currently, there are 56,614 patients waiting for a first adult Neurology appointment in the country. The Teleneurology Program at the *Hospital Las Higueras de Talcahuano* (HHT) was implemented in 2015 to help reduce both the number of patients waiting for a first consultation and their waiting times.

**Methods:**

This retrospective study analyzed a cohort of 8269 patients referred to the HHT Neurology clinic between 2013 and 2018, from primary, secondary, and tertiary health centers. Cox regression analyses were performed to determine the factors influencing each outcome (number of patients waiting for a consultation and waiting time), such as age, gender, referral health establishment and the type of consultation (whether in situ at the HHT or by synchronic telepresence through the Teleneurology Program).

**Results:**

Out of the 8269 patients included in the study, 1743 consulted the neurologist through the Teleneurology Program, while 6526 received a consultation in situ at the HHT. Since its implementation (2015) until the end of 2018, the Teleneurology program contributed to decrease the number of patients waiting for their first appointment from 3084 to 298. Waiting time for the first consultation was 60% shorter for patients enrolled in the Teleneurology program than those with consultation in situ at HHT (6.23 ± 6.82 and 10.47 ± 8.70 months, respectively). Similar differences were observed when sorting patient data according to the referral health center. Cox regression analysis showed that patients waiting for a traditional in situ first adult Neurology consultation at the HHT had a higher risk (OR = 6.74) of waiting 90% longer than patients enrolled in the Teleneurology Program, without significant differences due to gender or age.

**Conclusions:**

Data from this study show a significant contribution of the Teleneurology Program at the HHT to decrease the number of patients waiting for a first consultation with a neurologist, as well as shorter waiting times, when derived from primary and secondary health centers.

## Contributions to the literature

Since 2015, the Teleneurology Program at *Hospital Las Higueras de Talcahuano* has admitted adult Neurology patients. To evaluate the impact of Teleneurology on the population, we designed a retrospective study of 8269 patients from the hospital’s Neurology clinic. This article is the first to report a significant contribution of the Teleneurology Program to the decrease in the number and time of adult neurology patients waiting for a first appointment, when derived from primary and secondary health centers. *Research and data collection approved by the Ethics Committee of the Talcahuano Health Service (attached in Spanish).*

## Background

Chile is a South American country with 19,107,216 inhabitants as of 2019 [1], and a very particular continental geography of about 2650 miles (4270 km) in length, only 110 miles (175 km) in average width, and with the Pacific Ocean and the Andes Mountains naturally isolating its territory from the rest of the world. These characteristics that make the country a perfect touristic destination also hinder public transportation outside of its capital, Santiago. Taken together with a shortage of medical specialists, most of the tertiary health care is concentrated in Santiago and within the private health systems [1, 2]. As of December 2016, only 50% of adult neurologists in the country were affiliated to Public Health System [3], which provides healthcare to approximately 80% of the total population [4]. Thus, with 56,614 patients waiting for their first appointment as of June 30, 2019, Neurology ranks number 7 among the Chilean medical specialties with patients waiting for their first appointment [5].

Telemedicine implementation and development within the Chilean Public Health System arises as an opportunity to overcome both specialist shortage and transportation issues [6]. In the country, there are reported successes in the use of telemedicine for decreasing the number of patients waiting for a first consultation in Ophthalmology [7], Cardiology [8], Dermatology [9], and Orthopedics [10]. In contrast, no data has been reported for Teleneurology, as it appears to be only rarely implemented in some Chilean Public Health establishments [11, 12].

The Talcahuano Health Service (SST), located about 300 miles (500 km) south of Santiago, provides healthcare to 360,565 inhabitants of the region in 2019 [13]. The SST manages three hospitals, a Neurology Unit (NU) and a Telemedicine program (Teleprocess Unit), located at the *Hospital Las Higueras de Talcahuano* (HHT). The HHT Teleprocess Unit was created in 2008 as an asynchronous system, allowing storage and transfer of images, and consequently, remote clinical decision making. Since then, this Telemedicine program has allowed several landmarks: i) improvement in the prioritization of care for users with greater health risk, ii) improvement in the patient clinical registry, iii) efficient integration between the diverse professionals and technicians within a health team, and iv) contribution to the continuity in the follow-up and management of chronic patients [14].

The Teleneurology Program was created at the HHT Neurology Unit in March 2015 to help manage the 3084 adult neurological patients waiting for a first consultation. This program operates in synchronous mode, in which patients consult with a general practitioner trained for this purpose at their local health care establishment, in the telepresence of the neurologist specialist through an HDTV videoconference system [15]. This type of system has allowed the remote care of patients derived from primary and secondary local healthcare facilities [16, 17] as well as improved the flow of consultations of adult neurological patients, with very high user satisfaction [3, 15].

Despite, the positive experiences that have previously been reported, regarding significant improvements in the efficiency of patient care by telemedicine programs in other regions [10, 18, 19], evidence in Chilean teleneurology programs remain unpublished. Moreover, since the implementation of the Teleneurology Program, a seemingly decreasing trend of the number of patients waiting for their first consultation appears to be in contrast with the increasing trend in this figure for the rest of the country. Therefore, we set out to investigate the impact of the HHT Teleneurology Program on two main outcomes: the number of patients waiting for an adult neurology consultation and their waiting times. The present study is the first report of the effect of such a program improving both outcomes in Chile.

## Methods

### Description of the study population

For this retrospective cohort study, we analyzed data from patients referred to the HHT Neurology clinic, between January 2013 and December 2018, whether consulting in situ at the HHT or enrolled in the Teleneurology Program. Patients were referred from the following primary health facilities of the Public Health System: i) Community Family Health Centers (*CECOFS* for the Spanish acronym) *8 de Mayo*, *Cerro Estanque, Cosmito, El Santo, Esmeralda, España, Forjadores, Govinden, Libertad Gaete, Llafkelén, Los Lobos, Parque Central, Punta Parra, Rene Schneider,* and *Ríos de Chile,* and ii) Family Health Centers (*CESFAM* for the Spanish acronym) *Alberto Reyes, Bellavista, Hualpencillo, La Floresta, Leocan Portus, Lirquén, Los Cerros, Paulina Avendaño, Penco, San Vicente, Talcahuano Sur,* and *Posta Dichato.* Patients were also referred by a secondary health center (Mental Health Center, *COSAM* for the Spanish acronym) *Hualpén* and the tertiary health centers: HHT, *Tomé* Hospital, *Penco-Lirquén* Hospital, with the latter ending its participation in the program in 2018 (Additional file 1). Details of the modality of synchronous care of the Teleneurology consultation and the inclusion and exclusion criteria of the patients included in this study were previously described by Constanzo et al. [15].

### Variables

Main outcomes analyzed were:

i) Number of patients waiting for their first consultation, defined as the first care appointment of the patient with his or her medical practitioner, namely a neurologist in the present study, and.
ii) Waiting time for the first consultation (WT as acronym in Results section and tables), which refers to the time that elapses between the date when the patient requests an appointment and the date when the appointment takes place, measured in months. Follow-up and subsequent appointments are not included in the present study.

Independent variables analyzed were: *i)* age (60<, and > =60), *ii)* gender (male or female), *iii)* referral healthcare facility (primary, secondary, or tertiary), and *iv)* type of appointment (in situ at HHT or by telepresence through the Teleneurology Program). Finally, patients unnecessarily referrals come from patients referred to general practitioner for face-to-face attention in the HHT. These patients are registered in the system as poorly derived.

### Neurology medical team

The HHT Neurology Unit consists of 11 neurologists and two general practitioners. The neurologist group dedicate, as a group, 74 h per week to consultation in situ at HHT and 17 h per week to the Teleneurology Program. The general practitioners dedicate, as a group, 21 h per week for: *i)* coordination of care and follow-up of patients waiting for an appointment, *ii)* extension of chronic patient prescriptions, *iii)* follow-up of patients with a prolonged medical license or in the process of requesting disability pension. This allows the team of neurologists to focus exclusively on the resolution of patients’ neurological problems.

### Statistical analysis

The descriptive characteristics of the patients are shown as mean ± standard deviation and frequency (percentage), for continuous and categorical variables, respectively. A Kaplan-Meier test was carried out to evaluate the behavior of the WT outcome. Variables that could influence the WT outcome were analyzed through a Cox regression analysis. Results are presented as Odds Ratio (OR), and estimated coefficients (beta coefficients). Significance was set at *p* <  0.05. All analyses were performed with SPSS, version 25.

## Results

### Patient cohort description

This study analyzed retrospectively a cohort of 8269 adult outpatients who obtained their first appointment with a neurologist at the Neurology clinic of the *Hospital Las Higueras de Talcahuano* (HHT), between January 2013 and December 2018. Out of the total, 6526 patients (79%) consulted the specialist in situ at HHT and 1743 (21%) had their appointment with the telepresence of the neurologist, through the Teleneurology Program at HHT. As summarized, 2829 patients (34%) were male, while 5440 (66%) were female (Table 1). similar gender distribution percentages resulted after sorting patients in terms of the type of appointment (in situ or by telepresence).

**Table 1:** General descriptives of the patients treated by the HHT Neurology clinic, either in situ or through the Teleneurology program, Jan 2013 to Dec 2018

	Total Cohort *(n = 8269)*		Appointment in situ at the HHT *(n = 6526)*		Appointment through the Teleneurology Program *(n = 1753)*	
	*n*	%	*n*	%	*n*	%
Gender						
Male	2829	34	2265	35	564	32
Female	5440	66	4261	65	1179	67
Age group (years of age)						
15–30	1382	17	1164	18	218	13
31–45	946	11	705	10	241	14
46–60	1823	22	1422	22	401	23
Over 60	4118	50	3235	50	883	50
Referral healthcare facility						
Primary	5002	60	3411	52	1591	91
Secondary	42	1	42	1	0	0
Tertiary	3225	39	3073	47	152	9

On the other hand, 1382 (17%) patients were between 15 and 30 years of age, 946 (11%) were between 31 and 45 years old, 1823 (22%) were 46 and 60 years of age, and 4118 (50%) were over 60 years old. Similar age distribution was observed when grouping patients according to their type of appointment.

Finally, In situ neurologist attention at the HHT referred 3411 (52%) patients from primary health facilities, 42 (1%) from secondary health services, and 3073 (47%) from tertiary health care centers. The Teleneurology Program referred 1591 patients (91%) were from primary centers, and 152 (9%) from tertiary facilities, with no patients being referred from secondary services (Table 1).

### Number of patients waiting for their first neurology appointment

The Neurology clinic at HHT began the follow up of patients waiting for a first appointment with a specialist in March 2015. Table 2 shows the time course of the number of patients in this “waiting list” (for the Spanish legal term *“lista de espera”*) from March 2015 until December 2018. Out of the total 3084 patients waiting by March 2015, 743 (24%) were since 2013, 1740 (56%) since 2014, and 611 (20%) since the beginning of 2015. By December 2018, only 298 patients (90% lower than those in March 2015) were waiting for their appointment: 68 (23%) waiting since 2017 and 230 (80%) from 2018 (Table 2). Unfortunately, the patients from 2017 could be reached by the health service. Interestingly in certain cases, patients were unnecessarily referred to the Neurology clinic, due to misclassification of the symptoms. The clinic registry of declares declines from 105 patients referred under this situation in 2015, 82 in 2016, 29 in 2017, to 7 in 2018.

**Table 2:** Number of patients waiting for a first appointment with a neurologist at the Neurology clinic of the HHT

	Number of patients during each calendar year^a^						
Report date	2013	2014	2015	2016	2017	2018	2013–2018
01/03/2015	743	1730	611	–	–	–	3084
01/11/2015	9	1065	1401	–	–	–	2475
01/12/2015	–	907	1260	–	–	–	2167
01/02/2016	–	817	1205	91	–	–	2113
01/04/2016	–	711	1269	347	–	–	2327
01/05/2016	–	660	1105	99	–	–	1864
01/06/2016	–	583	1084	581	–	–	2248
01/09/2016	–	506	940	971	–	–	2417
01/10/2016	–	386	923	1035	–	–	2344
01/12/2016	–	–	640	1144	–	–	1784
01/01/2017	–	–	623	1490	38	–	2151
01/03/2017	–	–	530	1151	225	–	1906
01/06/2017	–	–	212	864	420	–	1496
01/07/2017	–	–	188	727	555	–	1470
01/09/2017	–	–	156	695	627	–	1478
01/11/2017	–	–	38	675	789	–	1502
01/12/2017	–	–	–	597	851	–	1448
01/01/2018	–	–	–	541	874	2	1417
01/02/2018	–	–	–	431	824	112	1367
01/03/2018	–	–	–	359	756	226	1341
01/04/2018	–	–	–	319	730	281	1330
01/05/2018	–	–	–	137	659	366	1162
01/06/2018	–	–	–	112	499	379	990
01/07/2018	–	–	–	92	236	417	745
01/09/2018	–	–	–	21	154	284	459
01/10/2018	–	–	–	–	113	226	339
01/11/2018	–	–	–	–	93	225	318
01/12/2018	–	–	–	–	68	230	298

^a^Data extracted from official reports from the Ministry of Health of Chile [20] for all patients of the cohort studied. It is of note that a group of patients are counted more than once among consecutive reports, for as long as they continue waiting for their appointment

### Waiting time for the first neurology appointment

To evaluate the possible effect that telemedicine may might had on the patient’s waiting time (WT) for their first Neurology appointment at the Neurology clinic of the HHT, WT data was distributed in 4 frames: up to 6 months, 7 to 10 months, 13 to 24 months, and over 24 months (Table 3). The average WT was 10.5 ± 8.7 months for patients consulting in situ at the HHT and 6.23 ± 6.82 months. Out of the total cohort analyzed in this study, 3845 patients (47%) waited for up to 6 months for their appointment, 944 (11%) waited between 7 and 12 months, 2618 (32%) waited between 13 and 24 months, and 862 (10%) waited for over 24 months, a distribution similar to that observed in the group of patients that consulted their neurologist in situ at the HHT (Table 3). In contrast, patients enrolled in the Teleneurology Program showed a different distribution, with 1053 (60%) waiting for up to 6 months, 228 (13%) waiting between 7 and 12 years, 346 (20%) waiting between 13 and 24 months, and 116 (7%) waiting for over 24 months (Table 3).

**Table 3:** Patient waiting time for their first appointment at the Neurology clinic of HHT

Waiting time frame (months)	Total Cohort *(n = 8269)*		Appointment in situ at the HHT *(n = 6526)*		Appointment through the Teleneurology Program *(n = 1753)*	
	*n*	%	*n*	%	*n*	%
Up to 6	3845	47	2792	43	1053	60
7–12	944	11	716	11	228	13
13–24	2618	32	2272	35	346	20
Over 24	862	10	746	11	116	7

**Fig. 1:**
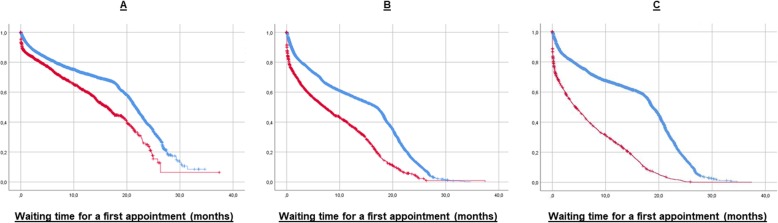
Kaplan-Meier survival curves of patient waiting time for a first appointment at the Neurology clinic of HHT. Data from patients who obtained their appointment *in situ* at the HHT (blue) and by telepresence through the Teleneurology Program (red) are shown for male patients (**a**), female patients (**b**); and patients referred from primary and secondary healthcare facilities (**c**) services

Kaplan-Meier survival analyses to better understand the putative effect of the Teleneurology program on patient WT, which resulted in the selection of 3 variables significantly showing an effect on the mean WT: male gender, female gender and patient referral from primary and secondary healthcare facilities (mean and median data summarized in Table 4). As depicted in Fig. 1, Kaplan-Meier function graphs show lower WT for patients who were enrolled in the Teleneurology Program compared to those who consulted their specialist in situ at the HHT. Male patients (panel A in Fig. 1) displayed significant difference in the median WT, with 21.7 ± 0.18 months (95% CI 21.3–22.0) for an in situ appointment, and 16.2 ± 0.60 months (95% CI 15.1–17.4) for an appointment with the telepresence of the specialist. A significant difference (*p* <  0.05) was also found in the median WT of female patients (panel B in Fig. 1), with 16.9 ± 0.29 months (95% CI 16.3–17.5) for an appointment in situ at HHT, and 7.2 ± 0.45 months (95% CI 6.3–8.1) when enrolled in the Teleneurology Program. The third variable which resulted with a significant median WT difference was the referral from primary or secondary healthcare facilities (panel C in Fig. 1), with 18.3 ± 0.15 months (95% CI 18.6–19.1) for in situ appointments and 4.4 ± 0.29 months (95% CI 3.8–5.0) for telepresence appointments. No differences were found when analyzing data according to any of the age groups or when patients were referred by tertiary healthcare services.

**Table 4:** Kaplan-Meier statistics of patients waiting for a first appointment at the Neurology clinic of HHT

Type of appointment	Waiting time (months)							
	Estimated Mean	SE	95% CI		Estimated Median	SE	95% CI	
			Lower	Upper			Lower	Upper
Male gender								
In situ	19.1	0.21	18.7	19.5	21.7	0.18	21.3	22.0
Teleneurology	15.4	0.68	14.1	16.7	16.2	0.60	15.1	17.4
Female gender								
In situ	14.0	0.13	13.7	14.2	16.9	0.29	16.3	17.5
Teleneurology	9.1	0.24	8.6	9.5	7.2	0.45	6.3	8.1
Referral from primary or secondary healthcare facilities								
In situ	15.7	0.14	15.4	16.0	18.9	0.15	18.6	19.1
Teleneurology	6.9	0.17	6.5	7.2	4.4	0.29	3.8	5.0

*SE* Standard error, *CI* Coefficient interval

Finally, a backward Cox regression analysis was carried out to understand the effect sizes of the 3 variables identified by the Kaplan-Meier analysis. As shown in Table 5, this analysis resulted in only one variable displaying a significant effect size (*p* <  0.001) on patient WT: “Type of Appointment”. This variable showed a beta coefficient of 1.91 ± 0.29 fold WT when it took place in situ at the HHT*,* as compared with patients consulting their neurologist by telepresence. This resulted in an odds ratio of 6.74 in the WT of patients with a traditional in situ appointment, compared to those enrolled in the Teleneurology Program (Table 5).

**Table 5:** Cox regression statistics of patients waiting for a first appointment at the Neurology clinic OF HHT

Step	Variable	Beta	SE	Wald	df	*P*	OR
1	Gender	−0.03	0.05	0.44	1.00	0.51	0.97
	Type of Appointment	1.91	0.08	504.84	1.00	< 0.001	6.75
	Age over 60 years old	0.08	0.05	2.64	1.00	0.10	1.08
2	Type of Appointment	1.91	0.08	504.63	1.00	< 0.001	6.74
	Age over 60 years old	0.08	0.05	2.79	1.00	0.09	1.08

Regression analysis was performed with the Backward method

*SE* Standard error, *df* x, *OR* Odds ratio

## Discussion

In Chile, the shortage of medical specialists added to the difficulties in the country’s geography give telemedicine an opportunity to improve access of patients to specialized healthcare. Mobility issues are of particular relevance to adult neurology patients due to many of the characteristic clinical manifestations of their illnesses. Telemedicine arises as an opportunity to improve access to specialized neurological care for patients who otherwise would have to their travel to tertiary healthcare facilities, where the specialists work.

By January 2015, the *Hospital Las Higueras de Talcahuano* (HHT), a tertiary healthcare center of the Chilean Public Health System located in the south of the country, had 3084 patients waiting for a first appointment with an adult neurology specialist, 743 of whom had been waiting since 2013. Thus, the institution created a Teleneurology Program for its Neurology clinic patients in March 2015, to improve their access to the specialist by way of an appointment with a general medical practitioner at their local primary healthcare facility and with the synchronous telepresence of the neurologist, located at HHT. The present study was aimed to evaluate the impact of such a program in both the number of patients waiting for a first appointment with a specialist in adult Neurology and the waiting time for the appointment to take place.

Data from this study show that there was a 90% decrease in the number of patients waiting for their first appointment with a neurologist between January 2015 and December 2018, which could have been contributed by the Teleneurology Program. Kaplan-Meier survival curves confirmed that patients enrolled in this program had to wait less time for their first appointment, as compared with patients that obtained a traditional in situ appointment, by 25% for males and 57% for females in the median waiting time. The difference in the effect between genders is likely to be rooted in cultural differences regarding patient compliance of Chilean females and males, characteristic in developing countries [21]; however, a size effect cannot be discarded due to the gender distribution of the cohort (over 65% female), which requires further research and it is out of the scope of the present study. On the other hand, the analysis also showed that patients referred to the Teleneurology Program from primary and secondary healthcare facilities had to wait 56% less time compared to those who had their appointment in situ at HHT. This result supports the idea that the program is taking charge of the burden of neurology patients particularly in areas far away from the tertiary healthcare facility where the neurologist is located, which are also the areas with the lower income within the population assigned to the HHT.

**Fig. 2:**
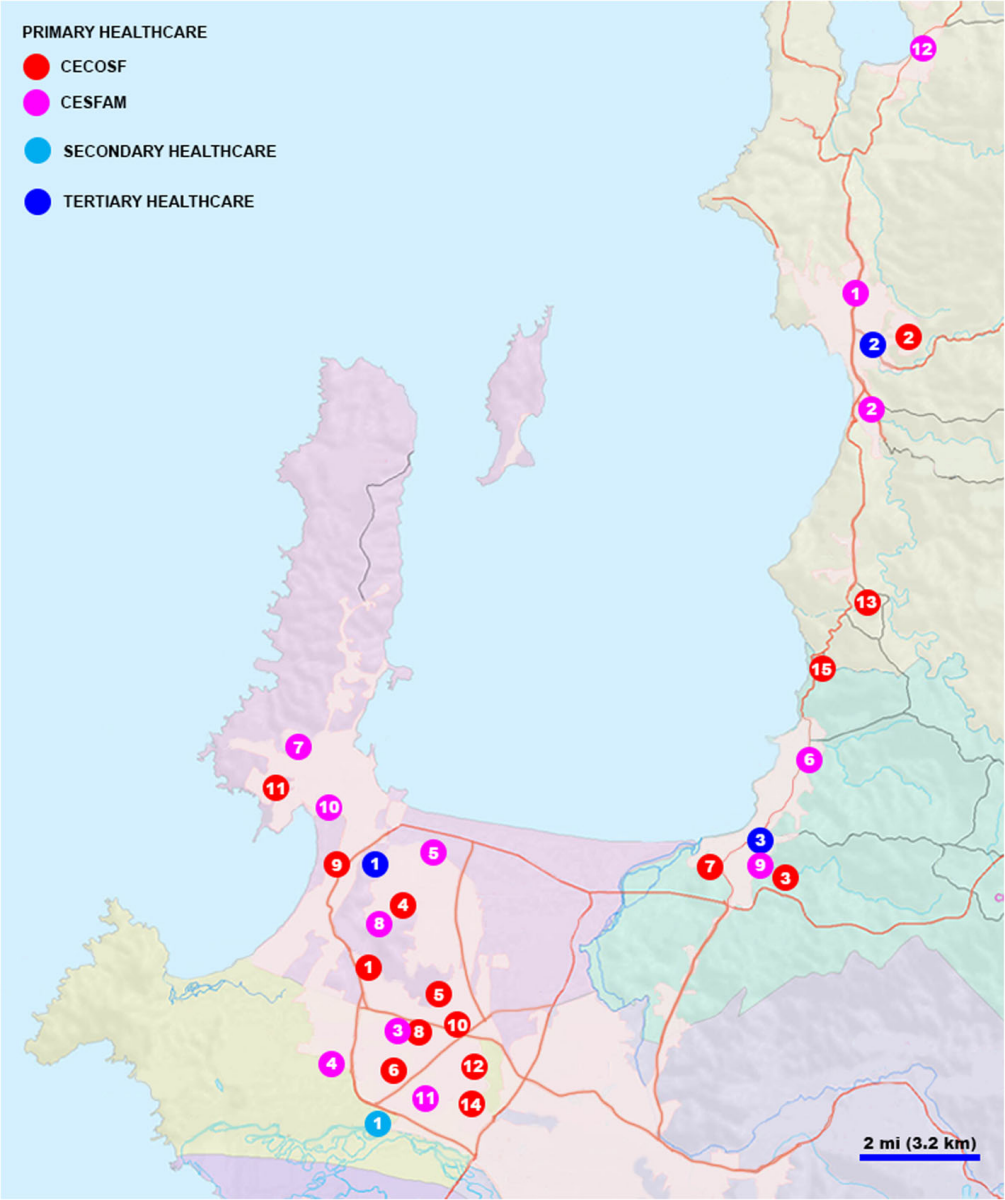
Geographical location of primary, secondary and tertiary healthcare facilities referring patients to the Neurology clinic at HHT

Further statistical analysis through Cox regression identified the Teleneurology Program with a significant effect size on patient waiting time for a first appointment with the specialist. In fact, a patient enrolled in the Teleneurology Program at HHT had to wait almost 7-fold less time for their appointment to take place. Therefore, the present study is the first to report an improvement of access to a neurology specialist in a tertiary healthcare facility with the use of a telemedicine-based system. It is possible that the success of the Teleneurology Program for patients referred from primary and secondary healthcare facilities is related to the higher feasibility of a neurology patient to travel to a local healthcare center compared to the tertiary service where the specialist work, which is directly related to the mobility issues affecting this population of patients in particular. However, similar results have been reported in Chile with synchronous telemedicine programs in other medical specialties, such as dermatology [22], ophthalmology [23], and orthopedics [10]. Therefore, further research is required to identify whether the travel distance of patients to the tertiary healthcare facility (Fig. 2) is a risk factor in the waiting time for a first appointment.

Taken together, results from this study support the idea that this type of telemedicine-based system can function as a screening and triage tool, contributing to solve the burden of out-patient neurological disease. In fact, the number of patients mistakenly referred to the Neurology clinic at HHT decreased 93% since the creation of the Teleneurology Program, supporting this idea. Recent studies in telehealth have described that one of the advantages of this health management system, in its ability to cover large areas of territory by improving accessibility [24, 25] and as has been shown in numerous studies, the most serious patients can be identified and referred for an in situ appointment, emergency or admittance as an in-patient [22, 26].

Overall, the Teleneurology Program at HHT appeared to contribute to the containment of the overwhelmed demand of in situ appointment, by covering patients of lower complexity according to clear criteria of reference and counter-reference. As in other latitudes, the Teleneurology Program at HHT improved the control and monitoring of neurological patients with clear and stable pathologies [27, 28]. The HHT Teleneurology system contributes significantly to the continuous education of primary health care doctors, neurology residents and neurologist doctors, by teaching them how the Teleneurology system works before beginning their functions in Teleneurology program, as well as, accompanying both professionals in the moment in which that each patient is treated, what is widely supported in the current literature [29–31].

Taking together with the recent report showing a high satisfaction perception by patients with access to this program [15], results from the present work strongly support the success of the Teleneurology Program at HHT in providing highly-accessible and satisfactory healthcare. Adoption of this type of programs by the Chilean Public Healthcare System would allow a consistent contribution to the country-wide decrease in both the number of patients waiting for a first appointment and the waiting time. This would entail: *i)* continuing medical education for all health personnel at different levels of care, *ii)* creation of protocols for resolving the most common pathologies, with appropriate reference and counter-referral criteria, *iii)* a good residence on-call schedule allowing the admittance of in-patients with severity criteria outside the scope of a Teleneurology protocol, *iv)* a high-quality neurological in-patient admittance service, allowing for study and therapy with clear standards and criteria, and *v)* efficient hierarchy of in situ care, including the support by general practitioners to unburden specialist with low complexity follow-ups, such as prescription refills. Currently, the Neurology unit at the HHT is extensively working in quality of care assurance, continuing education and looking forward to broadening their coverage of the neurological patient population in the region, due to an absence of patient in the waiting for a first consultation.

## Conclusions

Here we concluded that the HHT Teleneurology Program had a significant impact in reducing both outcomes: the number of patients waiting for an adult neurology consultation and their waiting times. We propose to continue this experience in other Chilean health centers, and to focus our efforts in the waiting list of control patients.

## Supplementary information

**Additional file 1.** Data based used in the present study.

## Data Availability

The dataset supporting the conclusions of this article is included within the article and its additional file.

## Abbreviations

SST: Health Service of Talcahuano of the Ministry of Health
HHT: Hospital las Higueras of Talcahuano
NU: Neurology Unit
